# Physical Mapping in a Triplicated Genome: Mapping the Downy Mildew Resistance Locus *Pp523* in *Brassica oleracea* L.

**DOI:** 10.1534/g3.111.001099

**Published:** 2011-12-01

**Authors:** Jorge D. Carlier, Claudia S. Alabaça, Nelson H. Sousa, Paula S. Coelho, António A. Monteiro, Andrew H. Paterson, José M. Leitão

**Affiliations:** * Center for Biodiversity, Functional & Integrative Genomics (BioFIG), FCT, Universidade do Algarve, Campus de Gambelas, 8005-139 Faro, Portugal; † Instituto Nacional de Recursos Biológicos, 2780-505 Oeiras, Portugal; ‡ Instituto Superior de Agronomia, Universidade Técnica de Lisboa, 1349-017 Lisboa, Portugal; § Plant Genome Mapping Laboratory, Departments of Crop and Soil Sciences, Plant Biology, and Genetics, University of Georgia, Athens, Georgia 30602, USA

**Keywords:** genetic resistance, plant disease resistance, map-based cloning, BAC contig, genome triplication

## Abstract

We describe the construction of a BAC contig and identification of a minimal tiling path that encompass the dominant and monogenically inherited downy mildew resistance locus *Pp523* of *Brassica oleracea* L. The selection of BAC clones for construction of the physical map was carried out by screening gridded BAC libraries with DNA overgo probes derived from both genetically mapped DNA markers flanking the locus of interest and BAC-end sequences that align to *Arabidopsis thaliana* sequences within the previously identified syntenic region. The selected BAC clones consistently mapped to three different genomic regions of *B. oleracea*. Although 83 BAC clones were accurately mapped within a ∼4.6 cM region surrounding the downy mildew resistance locus *Pp523*, a subset of 33 BAC clones mapped to another region on chromosome C8 that was ∼60 cM away from the resistance gene, and a subset of 63 BAC clones mapped to chromosome C5. These results reflect the triplication of the Brassica genomes since their divergence from a common ancestor shared with *A. thaliana*, and they are consonant with recent analyses of the C genome of *Brassica napus*. The assembly of a minimal tiling path constituted by 13 (BoT01) BAC clones that span the *Pp523* locus sets the stage for map-based cloning of this resistance gene.

Downy mildew caused by the oomycete *Hyaloperonospora brassicae* (Gäum.) (Göker *et al.* 2003) affects *Brassica oleracea* L. plants from seedlings in nurseries to adult plants in the field, reducing yield and severely compromising the quality of the marketable product. For some specific genotypes and environmental conditions, such as the Romanesco-type cauliflower in Brittany, losses due to this disease can even be total (Monot *et al.* 2010).

One of the most effective, low-cost, and ecologically benign methods for plant disease control is the use of genetically resistant plants. For downy mildew, several sources of genetic resistance have been identified at seedling and adult plant stages of *B. oleracea* (Natti and Atkin 1960; Natti *el al*. 1967; Dickson and Petzoldt 1993; Hoser-Krauze *et al.* 1995; Mahajan *et al.* 1995; Coelho *et al.* 1998; Jensen *et al.* 1999).

However, resistance to downy mildew in these two plant developmental stages is apparently determined by different genetic systems: plants that exhibit resistance at the cotyledonary phase can be susceptible at the adult phase and vice versa (Monteiro *et al.* 2005).

During the last few years, there were some advances in the genetic study of the inheritance of downy mildew resistance and in the isolation and cloning of resistance genes in *Brassica* species. One locus conferring downy mildew resistance at the cotyledon stage in broccoli (*Brassica oleracea* convar. *italica*) was genetically mapped by Giovannelli *et al.* (2002) and located in close linkage to the glucosinolate pathway gene *BoGsl-elong* on a dense map of *B. oleracea* (Gao *et al.* 2007). A second downy mildew resistance gene at seedling stage was recently mapped in Chinese cabbage (*Brassica rapa* ssp. *pekinensis*) (Yu *et al.* 2009).

A dominant and monogenically inherited resistance locus expressed at the adult plant stage was identified in broccoli by Coelho *et al.* (1998) and named *Pp523* (after a pathogen strain). This locus was later located on a new genetic map of RAPD and AFLP markers (Farinhó *et al.* 2004) within a linkage group assigned to the *B. oleracea* chromosome C8 (Carlier *et al.* 2011). Five DNA markers that defined a genomic region of 8.5 cM encompassing this resistance locus were then cloned, sequenced, and remapped as SCAR and CAPS markers. BLAST queries (www.ncbi.nihl.gov/blast) identified a genomic region syntenic to this *B. oleracea* genome segment at the extremity of the top arm of *Arabidopsis thaliana* L. chromosome 1 (Farinhó *et al.* 2007).

Map-based, or positional, cloning, is a common strategy for isolation of genes responsible for phenotypic differences. This strategy was used for the isolation of most of the >100 reference *R*-Genes so far included in the Plant Resistance Genes database (http://prgdb.cbm.fvg.it; Sanseverino *et al.* 2010). Map-based cloning, with specific variations, was also the central procedure used for the isolation of the *A. thaliana* genes *RPP5* (Parker *et al.* 1997), *RPP8* (McDowell *et al.* 1998), *RPP1* (Botella *et al.*, 1998), *RPP4* (Van Der Biezen *et al.* 2002), and *RPP2A/RPP2B* (Sinapidou *et al.* 2004), the single downy mildew resistance genes so far isolated in the Brassicaceae family.

One of the major steps in map-based cloning is the physical identification of the genomic region where the gene is located. For genomes still not fully sequenced, this implies the physical mapping of the gene of interest via construction of a contig of large insert DNA clones, usually BACs. Here we report the construction of a physical map of a genomic region of 2.9 cM that encompasses the downy mildew resistance locus *Pp523* in *B. oleracea*, carried out by exploiting the conserved synteny between *B. oleracea* and *A. thaliana* (Farinhó *et al.* 2007). One major obstacle to overcome was the triplicated nature of *B. oleracea* genome (O’Neill and Bancroft 2000; Lysak *et al.* 2005; Town *et al.* 2006).

## Material and Methods

### Plant material and DNA marker analyses

The *B. oleracea* mapping population (163 F2 plants), the evaluation of plant response to downy mildew, and the procedures for plant DNA extraction and molecular marker analyses have been previously described (Coelho and Monteiro 2003; Farinhó *et al.* 2004, 2007).

### BAC selection by overgo hybridization

Two gridded *B. oleracea* BAC libraries (BoT01 and BoCig) constructed at the Plant Genome Mapping Laboratory, University of Georgia, were used for identification of BAC clones located at the genomic region that spans the *Pp523* locus.

**Figure 1:**
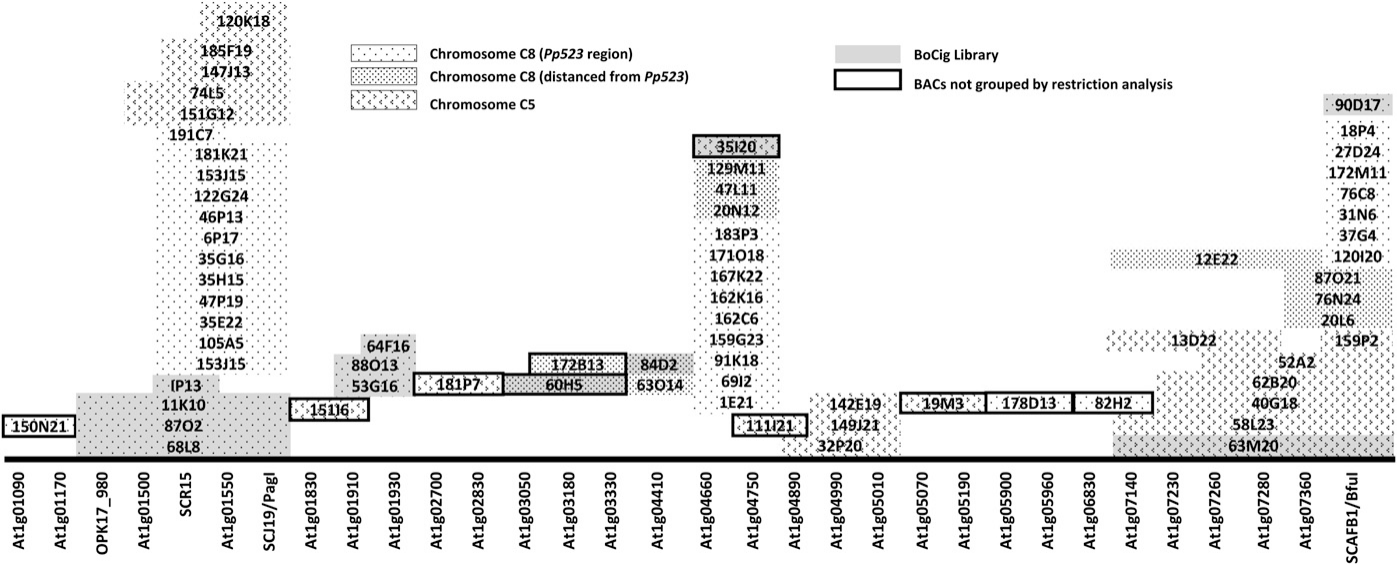
BAC clones (BoT01 and BoCig libraries) selected via hybridization against overgo probes derived from four *Brassica oleracea* markers that flank the locus *Pp523* and 28 loci of the corresponding syntenic genomic region of *Arabidopsis thaliana*.

Overgo probes hybridization analysis was carried out for markers OPK17_980, SCR15, SCJ19/PagI, and SCAFB1/BfuI, which define a 4.8 cM genomic region encompassing the *Pp523* locus (Farinhó *et al.* 2007), and for 28 *A. thaliana* sequences (At1g01090 to At1g07360; Figure 1 and File S1) within the syntenic region defined by the most external *B. oleracea* markers OPK.17_980 (At1g01220) and SCAFB1/BfuI (At1g07420). Sequences of 40 bp were selected within the DNA-marker sequences for design of 24 bp forward and reverse overgo primers, which shared an overlapping terminal sequence of 8 bp. Two overgo probes were designed for each marker sequence so that the forward primer of the first overgo and the reverse primer of the second overgo could generate a PCR product for confirmation of hybridizing BACs.

Overgo probes labeling was performed at 37° for 2 hr in a total volume of 15 μl containing 0.0067 nM forward and reverse primers denatured at 94° for 5 min and cooled on ice, 1 μg BSA, 2.5 U of Taq polymerase, 1 μl of [α^32^P]dATP (6000 Ci/mmol) (MP Biomedicals), 1 μl of [α ^32^P]dCTP (6000 Ci/mmol) (MP Biomedicals), and 3 μl OLB [oligo-labeling buffer without dATP or dCTP, and random hexamers (Ross *et al.* 1999)]. The labeled probes were filtered through Sephadex minicolumns to remove the unincorporated radioactive nucleotides.

Nylon membranes separated with a nylon mesh were incubated at 55° for 2 hr in a hybridization oven at 4.5 rpm in hybridization buffer [0.5 M sodium phosphate (pH 7.2), 7% (w/v) SDS, 1 mM EDTA, and 0.01% (w/v) BSA]. Radioactive overgo probes were hybridized at 55° for 18 hr, and membranes were washed thrice at 55° for 30 min with constant shaking, successively in buffer A [1x SSPE, 1% (w/v) SDS], buffer B [0.5 x SSPE, 1% (w/v) SDS], and again in buffer A. Membranes were blot-dried with filter paper, placed between two sheets of cellophane paper, and autoradiographed using two intensifying screens (L-Plus; Optonix) on X-ray film (Blue Medical, Source One) for 2 weeks at −80°.

The hits on X-ray films were scored manually using gridded transparent templates that were scanned and read by the software ABBYY FineReader 5.0. The hit scores were manually corrected and converted to individual BAC clone addresses using the BACEater software (http://bacman.sourceforge.net/program/BACEater.html).

The plasmid DNA of the selected BAC clones were isolated, the BAC ends were sequenced, and the sequences were submitted to GenBank.

### BAC fingerprinting (restriction analysis)

Plasmid DNA was isolated from BAC clones using a standard alkaline-lysis protocol and digested with 40 U of H*ind*III for 4.5 hr. The digestion products were run on 1% agarose gel electrophoresis for 16 hr at 95 V. The gel images were analyzed with IMAGE (Sulston *et al.* 1989), and the overlapping contigs were assembled using the software FPC V 4.7 and a cutoff E-value of e−7 (Soderlund *et al.* 2000).

### Selection of additional BAC clones

Two-hundred thirty additional *B. olerace*a BAC clones were selected *in silico* by exploiting the *B. oleracea*/*A. thaliana* syntenic relationship at the genomic region of the locus *Pp523*. The search for BAC-end sequences (BoT01 BAC library) exhibiting high level of similarity to *A. thaliana* sequences was performed using the *Brassica oleracea* BLAST search at the JCV Institute (http://blast.jcvi.org/er-blast/index.cgi?project=bog1) against 5000 nucleotides sequences consecutively retrieved from the *A. thaliana* chromosome 1 between At1g01770 and At1g07200 [Arabidopsis Information Resource (TAIR), www.arabidopsis.org/]. Sequence similarities with E-values greater than 0.001 were assumed nonsignificant (Table 1).

**Table 1:** Accurately mapped BAC clones

BAC	BLAST *A. thaliana*		Mapping		BAC	BLAST *A. thaliana*		Mapping		BAC	BLAST *A. thaliana*		Mapping	
	BAC–TF	BAC–TR				BAC–TF	BAC–TR				BAC–TF	BAC–TR		
**87O2***^a^*	At1g01190	NS	PCR	C8a	**167K22**	At1g04860	At1g04440	Map F	C8a	**172M11**	NS	At3g13445	PCR	C8a
**11K10***^a^*	NS	NS	Map F	C8a	**162C6**	At5g40170	At1g04470	Map F	C8a	**31N6**	NS	At1g07450	Map F.R	C8a
**68L8***^a^*	At1g25120	NS	Map R	C8a	**91K18**	At1g04470	At5g40170	Map R	C8a	**90D17***^a^*	Not Seq	At1g07390	Map R	C8a
**153J15**	At1g01600	At1g01230	PCR	C8a	**1E21**	At1g04540	At5g03380	Map R	C8a	**63O14**	At1g04300	At1g04510	Map F	C8b
**122G24**	At1g01600	NS	PCR	C8a	**183P3**	At1g04750	NS	PCR	C8a	**84D2***^a^*	NS	At1g04270	PCR	C8b
**35G16**	At1g01770	At1g01448	Map R	C8a	**162K16**	At1g04480	At5g40170	PCR	C8a	**47L11**	NS	At1g21060	Map F	C8b
**181K21**	At1g01610	Not Seq	PCR	C8a	**39E3**	Not Seq	At1g04160	PCR	C8a	**129M11**	Not Seq	Not Seq	PCR	C8b
**105A5**	TnLs	At1g01380	PCR	C8a	**107D22**	At1g04560	At3g03260	Map R	C8a	**20N12**	NS	At1g21060	PCR	C8b
**35H15**	At1g01770	At1g01448	PCR	C8a	**33N5**	At1g04540	At1g05136	Map R	C8a	**12E22**	At1g07450	At1g07120	PCR	C8b
**46P13**	At1g01770	NS	PCR	C8a	**159G23**	At1g04550	NS	Map R	C8a	**87O21**	At1g07480	TnLs	PCR	C8b
**6P17**	At1g01770	NS	PCR	C8a	**171O18**	At1g04560	NS	PCR	C8a	**63M20***^a^*	At1g07460	Not Seq	Map F	C8b
**161N21**	Not Seq	Not Seq	PCR	C8a	**49K12**	At1g05370	At1g05180	Map R	C8a	**76N24**	TnLs	NS	PCR	C8b
**35E22**	Not Seq	TnLs	PCR	C8a	**83K19**	At1g05180	At1g05470	Map F	C8a	**20L6**	NS	NS	PCR	C8b
**47P19**	TnLs	TnLs	PCR	C8a	**85O24**	At1g05180	NS	PCR	C8a	**150N21**	Not Seq	Not Seq	PCR	C5
**191C7**	Not Seq	Not Seq	PCR	C8a	**111O21**	At1g05470	At1g05230	Map R	C8a	**185F19**	NS	At1g01460	Map R	C5
**1P13***^a^*	Not Seq	Not Seq	PCR	C8a	**15N10**	At1g05310	At1g05510	PCR	C8a	**120k18**	At1g01410	NS	PCR	C5
**53G16***^a^*	NS	At1g02100	PCR	C8a	**9O6**	At1g05440	At1g05590	PCR	C8a	**74L5**	NS	NS	PCR	C5
**88O13***^a^*	At1g01820	TnLs	PCR	C8a	**2M20**	At1g05230	At1g05510	PCR	C8a	**151G12**	NS	TnLs	Map R	C5
**64F16***^a^*	Not Seq	Not Seq	PCR	C8a	**101N4**	At1g05200	NS	PCR	C8a	**147J13**	Not Seq	NS	PCR	C5
**19M21**	At2g48090	At1g02070	Map R	C8a	**63E7**	At1g05230	NS	PCR	C8a	**151I6**	Not Seq	At1g01970	Map R	C5
**117M5**	At2g48140	At1g01950	Map F	C8a	**115C6**	At2g32300	At2g32010	PCR	C8a	**181P7**	At1g02570	At1g02860	Map F	C5
**65H5**	At1g01950	At1g02205	PCR	C8a	**23K23**	NS	At1g05950	PCR	C8a	**35I20***^a^*	At2g25440	At1g04560	Map R	C5
**65L14**	At1g02010	NS	Map F	C8a	**24H17**	TnLs	At1g05950	PCR	C8a	**142E19**	NS	At1g05020	Map R	C5
**98F7**	At2g48140	At1g01960	PCR	C8a	**178D13**	NS	At1g06130	Map F	C8a	**32P20**	At1g04840	At5g40170	Map F	C5
**96L11**	TnLs	At1g02580	Map R	C8a	**121A8**	NS	At1g05630	Map R	C8a	**111I21**	At1g04650	At1g04910	Map R	C5
**104H17**	At1g02660	At1g02230	PCR	C8a	**28N8**	NS	At1g70920	PCR	C8a	**18G3**	At1g05020	At1g04840	PCR	C5
**97K22**	At1g02750	NS	PCR	C8a	**117B1**	At1g06490	NS	PCR	C8a	**149J21**	Not Seq	Not Seq	PCR	C5
**53O21**	At1g02660	NS	PCR	C8a	**114F8**	NS	At1g06590	PCR	C8a	**19M3**	At1g05030	At1g05230	Map F	C5
**6D7**	NS	At1g02270	Map F	C8a	**11A22**	At1g06680	NS	PCR	C8a	**89C6**	At1g05577	At1g05690	PCR	C5
**92O1**	NS	At1g02990	PCR	C8a	**38E20**	At1g06490	NS	PCR	C8a	**16F14**	At1g05577	NS	Map F	C5
**68M7**	At1g03010	NS	PCR	C8a	**84E23**	At1g07110	NS	PCR	C8a	**54F20**	At1g06510	At1g06270	Map R	C5
**97P4**	NS	NS	PCR	C8a	**88A18**	NS	At1g06780	Map R	C8a	**90L6**	At1g06510	At1g06780	Map F	C5
**97K11**	At4g33910	At1g02980	PCR	C8a	**82H2**	At1g06740	NS	PCR	C8a	**13D22**	TnLs	NS	Map F	C5
**10C19**	At1g03080	At1g03010	Map R	C8a	**6K18**	At1g07110	At1g07080	Map R	C8a	**58L23**	NS	At1g07420	PCR	C5
**7L14**	At1g03140	NS	Map F	C8a	**51P12**	At1g06930	NS	Map F	C8a	**159P2**	Not Seq	Not Seq	PCR	C5
**111P15**	NS	At1g03475	PCR	C8a	**76C8**	At1g07510	At1g07200	PCR	C8a	**62B20**	NS	At1g07230	Map R	C5
**13N3**	NS	At1g03890	Map R	C8a	**18P4**	NS	At1g07200	PCR	C8a	**52A2**	At1g07570	At1g07260	PCR	C5
**40J10**	At1g04210	NS	PCR	C8a	**27D24**	At1g07485	At1g07250	PCR	C8a	**40G18**	At1g07230	TnLs	PCR	C5
**112O12**	At1g04470	At1g04210	PCR	C8a	**120I20**	NS	At1g07570	PCR	C8a					
**69I2**	At1g04440	At1g04750	Map R	C8a	**37G4**	At1g07560	At3g13445	PCR	C8a					

TnLs, transposable element-like sequence; NS, not significant similarity; Not Seq, not sequenced; PCR, anchored by PCR; Map F, mapped forward BAC end; Map R, mapped reverse BAC end; TF, forward terminus; TR, reverse terminus; C8a, chromosome C8 (*Pp523* region); C8b, chromosome C8 (second mapping region); C5, chromosome C5.

a BoCig library.

### Genetic and physical mapping of BAC clones

Specific primers were designed to convert BAC-end sequences into sequence-tagged site (STS) markers (see File S1). Polymorphic (BAC-end–derived) STS markers were genetically mapped using the JoinMap 3.0 software (Van Ooijen and Voorrips 2001) set for the Kosambi function. The same software was used for drawing the linkage groups. Both polymorphic and monomorphic STS markers were used to establish and stabilize the physical map establishing the BAC-to-BAC ligation by PCR. The PCR products of the anchorage points between the BAC clones of the minimal tiling path were sequenced to confirm their similarity to the original BAC-end sequence.

## Results

The screening of BoT01 and BoCig BAC libraries resulted in the identification of 58 BoT01 BAC clones and 12 BoCig BAC clones (Figure 1), putatively surrounding the downy mildew resistance gene.

The fingerprinting (restriction) analysis of this set of BAC clones allowed their grouping into 11 small groups of at least two overlapping clones; nine BAC clones remained ungrouped (Figure 1). The BAC clones were assembled into a putative contig following the linear order of the Arabidopsis loci used to design the overgo probes (Figure 1).

**Figure 2:**
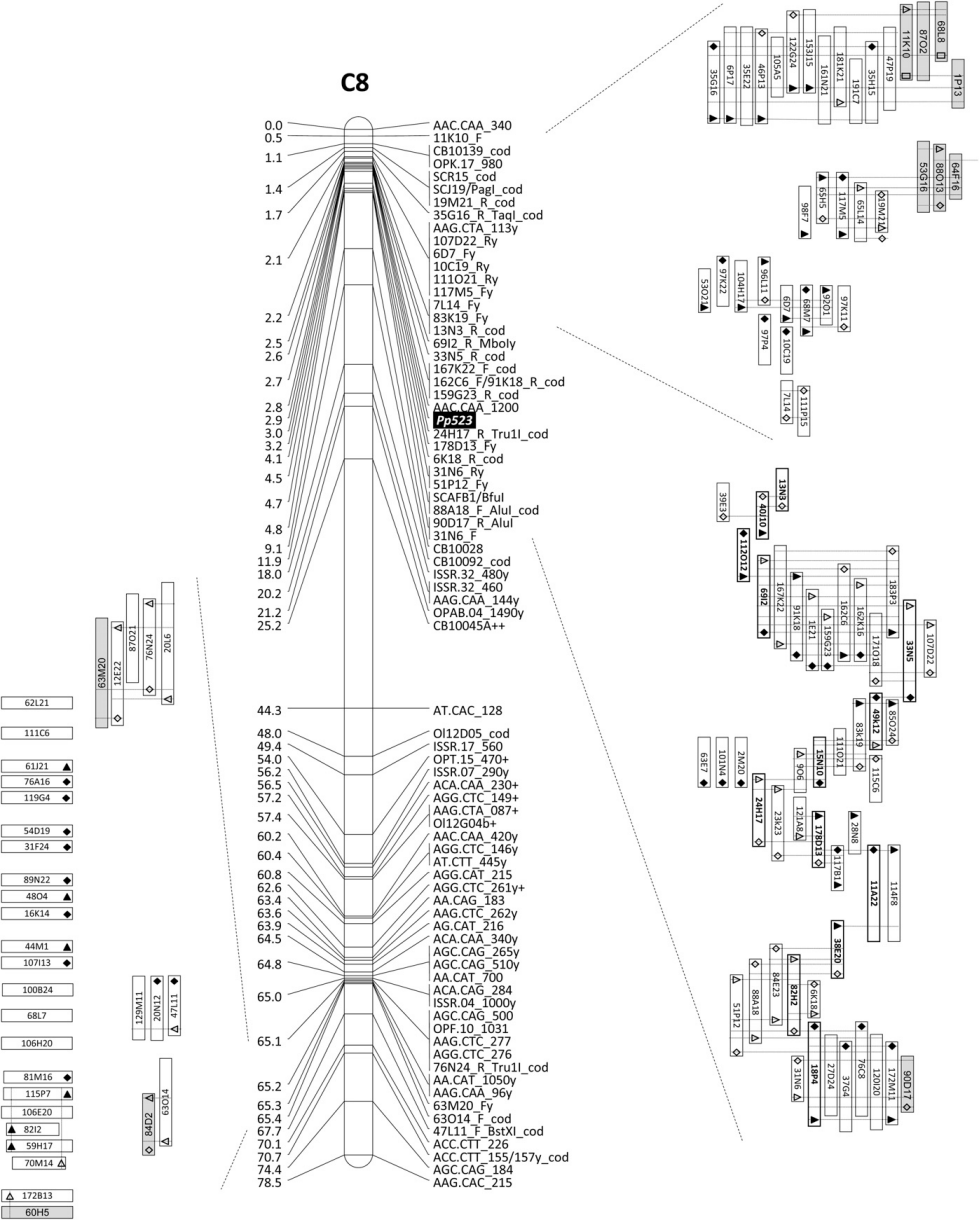
BAC clones mapped in chromosome C8. (Right) BAC clones mapped near the locus *Pp523*. (Left) BAC clones mapped apart from the resistance locus. Accurately mapped clones are represented vertically. Premapped clones are represented horizontally, ordered according to their collinearity with *A. thaliana*. The forward and reverse end of BAC clones are represented by a triangle and a lozenge, respectively. Black-filled triangles and lozenges indicate sequence identity between overlapping BAC ends. BAC-to-BAC (PCR) ligations are indicated by intersecting dotted lines.

**Figure 3:**
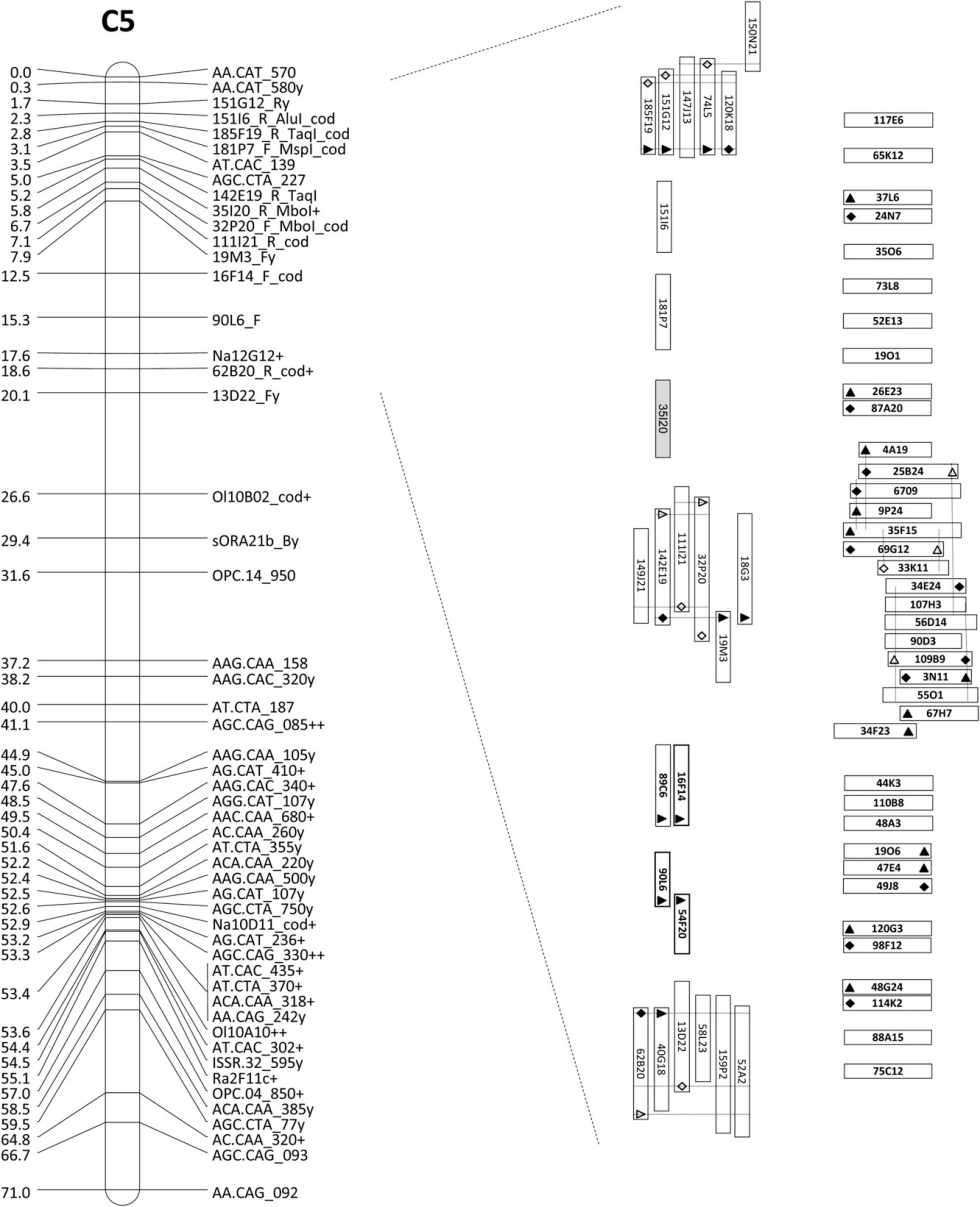
BAC clones mapped in chromosome C5. Accurately mapped BAC clones are displayed vertically. Premapped BAC clones are presented horizontally, ordered according to their collinearity with *A. thaliana*. BAC-to-BAC ligations (via PCR) and BAC-end identification are represented as in Figure 2.

Excluding the cases of absence of significant similarity and of similarity to transposable element-like sequences, the order of the end sequences of these BAC clones appeared collinear to the *A. thaliana* genome (Table 1). However, genetic mapping associated the BAC clones with three different genomic regions of *B. oleracea*: a) some mapped as expected near the locus *Pp523* in chromosome C8; b) a second, relatively smaller group mapped in the same chromosome but ∼60 cM away from *Pp523*; and c) a relatively large third group of BAC clones mapped in chromosome C5 (Figures 2 and 3).

The genetic mapping of the second set of BAC clones (selected *in silico*) from the BoT01 library followed the same tendency as the first set, mapping to the same three regions of the *B. oleracea* genome (Figures 2 and 3).

Once this problem was identified, a premapping step was included based on the segregation analysis of 14 progeny plants and subsequently confirming the segregation analysis of the putative *Pp523*-related clones in the remaining mapping population. As the main objective of this work was the construction of a BAC contig spanning the resistance gene of interest, the accurate mapping of some BAC clones in the second genomic region (in chromosome C8) and in the third genomic region (in chromosome C5) was not done.

The BAC clones that remained at the premapped stage are clearly discriminated (horizontally displayed) in Figures 2 and 3 and File S1.

Multiple BAC clones were anchored to the genetically mapped ones, either by inferring overlap (established by restriction analysis and confirmed by PCR), or by BAC-to-BAC ligation through PCR (using STS markers derived from BAC-end sequences), or in some cases, by alignment of identical end sequences. Anchored BAC clones were accepted as being genetically mapped, and they are displayed vertically in the above-cited figures.

In total, 83 BAC clones were accurately mapped in the region (∼4.6 cM in the present map) surrounding the downy mildew resistance locus *Pp523* in chromosome C8 (Figure 2). A relatively smaller group of 33 BAC clones were mapped at the other end of the chromosome C8 (Figure 2), while a large group of 63 BAC clones mapped to chromosome C5, where they are scattered throughout 18.5 cM (Figure 3).

The distribution of the selected BAC clones by more than one location was not completely surprising because the triplicate nature of Brassica genomes has been extensively documented both at the genetic map (Cavell *et al.* 1998; Lagercrantz 1998; Parkin *et al.* 2005) and the microsynteny levels (O’Neill and Bancroft 2000). The triplication of the Brassica genomes, despite multiple chromosome rearrangements, gene loss, and insertions (Town *et al.* 2006), is accompanied by extensive conservation of macro- and microsynteny (Kowalski *et al.* 1994; Lan *et al.* 2000; O’Neill and Bancroft 2000; Parkin *et al.* 2005; Kaczmarek *et al.* 2009) with *A. thaliana*, a feature that seems also to be valid for the genomic region that surrounds the *Pp523* locus in *B. oleracea*.

**Figure 4:**
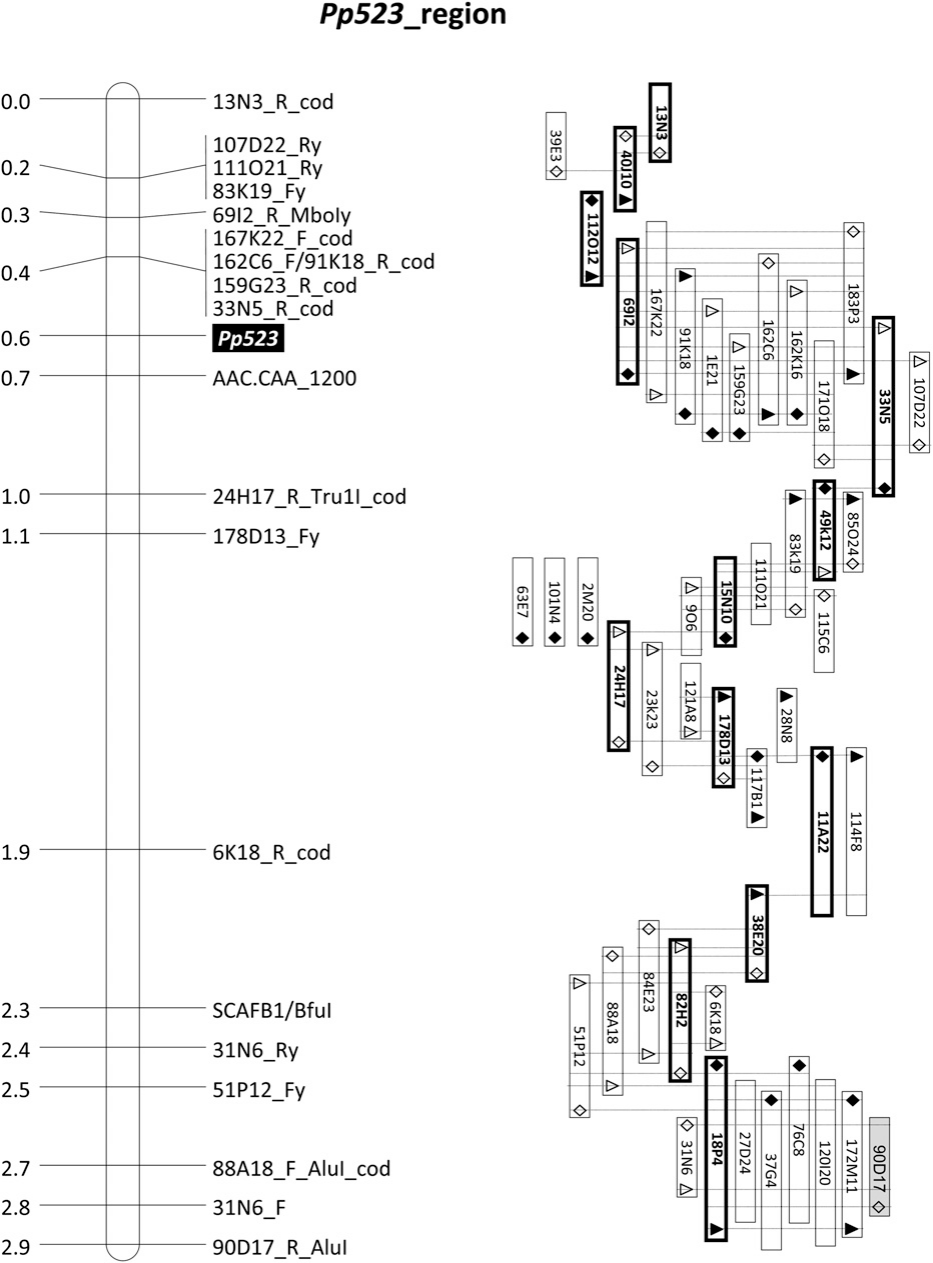
Genetic and physical map of the genomic region that encompasses the downy mildew resistance locus *Pp52*3. Bold outline and bold text identify 13 BAC clones (BoT01 library) that constitute a minimal tiling path in the physical map. Triangles, lozenges, and dotted lines are as in Figures 2 and 3.

A fine genetic map of the 4.8 cM region that encompasses locus *Pp523* was assembled by the inclusion of 25 new STS markers derived from BAC-end sequences (Figure 2). This allowed defining a shorter genomic region of ∼2.9 cM spanning the downy mildew resistance locus *Pp523* for the construction of a robust physical map for which a minimal tiling path of 13 BAC clones (BoT01 library) was established (Figure 4). Because of possible errors due to the triplication of the genome, which can originate multiple PCR products similar in length but with relatively different sequences and from different genomic loci, the PCR products that confirm the BAC-to-BAC linkages within the minimal tiling path were sequenced and carefully compared with the original BAC-end (STS) sequences used to design the primers. In all cases, they were identical.

## Discussion

Exploitation of the genetic similarity and syntenic relationship between *A. thaliana* and *B. oleracea* has guided the construction of a physical map surrounding the downy mildew resistance locus *Pp523*, by integration of genetic mapping with probe hybridization to BAC libraries and *in silico* selection of BAC clones using end-sequence information.

Two main obstacles have slowed, but not compromised, the accomplishment of this task: a) the large amount of transposable element-like sequences in the *B. oleracea* genome; and 2) the triplicate nature of the *B. oleracea* genome.

A large percentage (62 out of 429; 14.5%) of the BAC clones of the BoT01 library exhibit transposable element-like sequences at one or at both ends. By creating artifactual similarities between BAC-end sequences and between these and Arabidopsis genome sequences, this genome feature significantly reduced the number of BAC-end sequences suitable for mapping purposes and constrained our ability to employ Brassica/Arabidopsis synteny. The total length of transposable elements in *B. oleracea* has been calculated to be ∼15 times that of *A. thaliana* and to represent ∼120 Mb or 20% of the genome, leading to the suggestion that amplification of RNA and DNA transposable elements significantly contributed to the genome expansion of this crop species (Zhang and Wessler, 2004).

Nevertheless, the triplication of the genomic region of interest was the major constraint to a more efficient exploitation of the *A. thaliana*/*B. oleracea* genetic relatedness during the construction of the present physical map.

Besides the region in the *B. oleracea* chromosome C8 where the *Pp523* locus was previously mapped (Farinhó *et al.* 2004, 2007; Carlier *et al.* 2011), the BAC clones mapped in two additional regions, one at ∼60 cM in the same chromosome (C8) and another, apparently larger, in chromosome C5, evidencing a triplication of this Arabidopsis genomic region in *B. oleracea*. Today is largely accepted that the diploid *Brassica* species are paleohexaploids (Schmidt *et al.* 2001; Parkin *et al.* 2003; Lysak *et al.* 2005). With the support of various other studies that highlighted the Brassica genome triplication (Cavell *et al.* 1998; Lagercrantz 1998; Lan *et al.* 2000; O’Neill and Bancroft 2000; Parkin *et al.* 2005) and their own data, Lysak *et al.* (2005) proposed that after the Arabidopsis and Brassica lineages split, ∼14–24 Mya (millions of years ago) according to Yang *et al.* (1999) and Koch *et al.* (2000), an hexaploidation event occurred 7.9–14.6 Mya that gave rise to an ancestral triplicated *Brassiceae* genome, a feature that remained distinctive of all species of this tribe.

The early findings of Kowalski *et al.* (1994) and the comparative genetic mapping of over one thousand RFLP loci in *A. thaliana* and *B. napus* carried out by Parkin *et al.* (2005) suggested the existence of ∼20–25 conserved genomic units within the *A. thaliana* genome which duplication and rearrangement could generate the present *B. napus* genome. The majority of the conserved units were found in six copies, and 81% of the loci used for comparison were mapped to the triplicated regions by Parkin *et al.* (2005), consistent with the hypothesis of a hexaploid ancestor for the diploid Brassica progenitors. Nevertheless, the mechanism of formation of the present structure of the Brassica genomes is assumed to include multiple rearrangements via insertions, deletions, and translocations (Parkin *et al.* 2005; Town *et al.* 2006). The comparative mapping study of Parkin *et al.* (2005) and, specifically, the block of markers A (C1A) at the terminus of the top arm of *A. thaliana* chromosome 1 identified by these authors are of particular interest. This block corresponds to the genome block A defined by Schranz *et al.* (2006) in the “ancestral karyotype” of Lysak *et al.* (2006), which is delimited by the *A. thaliana* sequences At1g01560 and At1g19330, clearly spanning the *A. thaliana* genome segment between loci At1g01570 and At1g07420 syntenic to the *Pp523* region enclosed by the homologous *B. oleracea* markers SCJ19/PagI and SCAFB1/Bfu (Farinhó *et al.* 2007). This genome block (C1A or A) was found by Parkin *et al.* (2005) to have: i) a counterpart in the extremity of the linkage group/chromosome N18 (C8) apparently corresponding to the *B. oleracea* chromosome C8 region where the downy mildew resistance gene *Pp523* is embedded and part of the selected BAC clones map to (in the present work); ii) a second homologous region in the same chromosome (N18/C8), which apparently corresponds to the second region of BAC mapping; and iii) a large homologous region in the chromosome (N15/C5) corresponding to the *B. oleracea* third genome region to which a large group of the BAC clones also map. No other counterparts for this Arabidopsis genomic C1A/A segment were identified among the other *B. napus* C genome chromosomes (N11–N19).

The analysis of an integrated map of *B. napus* that includes the map of Parkin *et al.* (2005) recently published by Wang *et al.* (2011) allows the above observations to be clearly confirmed, as this map shares common reference SSR markers with our map (Carlier *et al.* 2011). Nevertheless, note that chromosome C8 of our map and those of Wang *et al.* (2011) and Parkin *et al.* (2005) are inverted relative to one another. The analysis of the alignment of *B. napus* markers with their homology BLAST hits within the Arabidopsis chromosomes (Wang *et al.* 2011) shows that the C1A/A block presents two main concentration plots of collinear hits in opposite directions at the expected positions on chromosome C8 and a large third concentration plot of hits on chromosome C5. Some hits can be observed on chromosome C7, whereas the other C genome chromosomes exhibit almost no hits. These results coincide and are confirmed by our BAC mapping results. Except for 2 BACs mapped to chromosome 2 and 1 BAC mapped to chromosome C6, the other (179) BACs mapped to two different regions on chromosome C8 and one region on chromosome C5.

One might expect the levels of identity between the *B. oleracea* BAC-end sequences and a specific Arabidopsis DNA sequence to exhibit some kind of pattern or tendency according to the Brassica genome region where they map. However, this is not the case. For example, the BAC-end sequences 49K12TR (C8, *Pp523* region), 106H20TR (C8, distant from *Pp523*), and 19M3TF (C5) show, respectively, 94%, 87%, and 90% of identity to a sequence stretch of gene At1g05180, whereas the BAC ends 121A8TR (C8, *Pp523* region), 76A16TR (C8, distant from *Pp523*), and 89C6TF (C5) show, respectively, 85%, 87%, and 90% identity to a sequence stretch of gene At1g05630. In other words, the location of a specific DNA sequence in the *B. oleracea* genome cannot be inferred from its level of identity to a specific *A. thaliana* sequence.

So far, a 2.0x BAC genome library from a downy mildew resistant S4 line derived from the original resistant genotype has been constructed at the University of Algarve, and a replica of the minimal tiling path (Figure 4) is currently being assembled using this BAC library. The identification of polymorphisms between the two BAC contigs, in particular regarding disease resistance gene-like sequences, is expected to produce significant information to foster our research toward the isolation of the downy mildew resistance gene *Pp523*.

## Supplementary Material

Supporting Information

## References

[bib1] Botella M. A. Parker J. E. Frost L. N. Bittner-Eddy O. D. Beynon J. L., 1998 Three genes of the Arabidopsis RPP1 complex resistance locus recognize distinct *Peronospora parasitica* avirulence determinants. Plant Cell 10: 1847–1860 9811793 10.1105/tpc.10.11.1847 PMC143951

[bib2] Carlier J. D. Alabaça C. A. Coelho P. S. Monteiro A. A. Leitão J. M., 2011 The downy mildew resistance locus *Pp523* is located on chromosome C8 of *Brassica oleracea* L. Plant Breeding (DOI: 10.1111/j.1439-0523.2011.01904.x) 10.1534/g3.111.001099 PMC3276173 22384370

[bib3] Cavell A. C. Lydiate D. J. Parkin I. A. Dean C. Trick M., 1998 Collinearity between a 30-centiMorgan segment of *Arabidopsis thaliana* chromosome 4 and duplicated regions within the *Brassica napus* genome. Genome 41: 62–69 9549059

[bib4] Coelho P. S. Leckie D. Bahcevandziev K. Valério L. Astley D., 1998 The relationship between cotyledon and adult plant resistance to downy mildew (*Peronospora parasitica*) in *Brassica oleracea*. Acta Hortic. 459: 335–342

[bib5] Coelho P. S. Monteiro A. A., 2003 Inheritance of downy mildew resistance in mature broccoli plants. Euphytica 131: 65–69

[bib6] Dickson M. H. Petzoldt R., 1993 Plant age and isolate source affect expression of downy mildew resistance in broccoli. HortScience 28: 730–731

[bib7] Van Der Biezen E. A. Freddie C. T. Kahn K. Parker J. E. Jones J. D., 2002 Arabidopsis *RPP4* is a member of the *RPP5* multigene family of TIR-NB-LRR genes and confers downy mildew resistance through multiple signalling components. Plant J. 29: 439–451 11846877 10.1046/j.0960-7412.2001.01229.x

[bib8] Farinhó M. Coelho P. Carlier J. Svetleva D. Monteiro A., 2004 Mapping of a locus for adult plant resistance to downy mildew in broccoli (*Brassica oleracea* convar. *italica*). Theor. Appl. Genet. 109: 1392–1398 15278283 10.1007/s00122-004-1747-0

[bib9] Farinhó M. Coelho P. Monteiro A. Leitão J., 2007 SCAR and CAPS markers flanking the *Brassica oleracea* L. *Pp523* downy mildew resistance locus demarcate a genomic region syntenic to the top arm end of *Arabidopsis thaliana* L. chromosome 1. Euphytica 157: 215–221

[bib10] Gao M. Li G. Yang B. Qiu D. Farnham M., 2007 High-density *Brassica oleracea* linkage map: identification of useful new linkages. Theor. Appl. Genet. 115: 277–287 17592603 10.1007/s00122-007-0568-3

[bib11] Göker M. Voglmayr H. Riethmüller A. Weiß M. Oberwinkler F., 2003 Taxonomic aspects of Peronosporaceae inferred from Bayesian molecular phylogenetics. Can. J. Bot. 81: 672–683

[bib12] Giovannelli J. L. Farnham M. W. Wang M., 2002 Development of sequence characterized amplified region markers linked to downy mildew resistance in broccoli. J. Am. Soc. Hortic. Sci. 127: 597–601

[bib13] Hoser-Krauze J. Lakowska-Ryk E. Antosik J., 1995 The inheritance of resistance of some *Brassica oleracea* L. cultivars and lines to downy mildew, *Peronospora parasitica* (Pers) ex. Fr. J. Appl. Genet. 36: 27–33

[bib14] Jensen B. D. Hockenhull J. Munk L., 1999 Seedling and adult plant resistance to downy mildew (*Peronospora parasitica*) in cauliflower (*Brassica oleracea* convar *botrytis* var *botrytis*). Plant Pathol. 48: 604–612

[bib15] Kaczmarek M. Koczyk G. Ziolkowski P. A. Babula-Skowronska D. Sadowski J., 2009 Comparative analysis of the *Brassica oleracea* genetic map and the *Arabidopsis thaliana* genome. Genome 52: 620–633 19767893 10.1139/G09-035

[bib16] Koch M. A. Haubold B. Mitchell-Olds T., 2000 Comparative evolutionary analysis of chalcone synthase and alcohol dehydrogenase loci in Arabidopsis, Arabis, and related genera (Brassicaceae). Mol. Biol. Evol. 17: 1483–1498 11018155 10.1093/oxfordjournals.molbev.a026248

[bib17] Kowalski S. D. Lan T.-H. Feldmann K. A. Paterson A. H., 1994 Comparative mapping of *Arabidopsis thaliana* and *Brassica oleracea* chromosomes reveals islands of conserved gene order. Genetics 138: 499–510 7828831 10.1093/genetics/138.2.499 PMC1206166

[bib18] Lagercrantz U., 1998 Comparative mapping between *Arabidopsis thaliana* and *Brassica nigra* indicates that *Brassica* genomes have evolved through extensive genome replication accompanied by chromosome fusions and frequent rearrangements. Genetics 150: 1217–1228 9799273 10.1093/genetics/150.3.1217 PMC1460378

[bib19] Lan T.-H. DelMonte T. A. Reischmann K. P. Hyman J. Kowalski S. P., 2000 An EST-enriched comparative map of *Brassica oleracea* and *Arabidopsis thaliana*. Genome Res. 10: 776–788 10854410 10.1101/gr.10.6.776 PMC310908

[bib20] Lysak M. A. Koch M. A. Pecinka A. Schubert I., 2005 Chromosome triplication found across the tribe *Brassiceae*. Genome Res. 15: 516–525 15781573 10.1101/gr.3531105 PMC1074366

[bib21] Lysak M. A. Berr A. Pecinka A. Schmidt R. McBreen K., 2006 Mechanisms of chromosome number reduction in *Arabidopsis thaliana* and related Brassicaceae species. Proc. Natl. Acad. Sci. USA 103: 5224–5229 16549785 10.1073/pnas.0510791103 PMC1458822

[bib22] Mahajan V. Gill H. S. More T. A., 1995 Inheritance of downy mildew resistance in Indian cauliflower (group III). Euphytica 86: 1–3

[bib23] McDowell J. M. Dhandaydham M. Long T. A. Aarts M. G. Goff S., 1998 Intragenic recombination and diversifying selection contribute to the evolution of downy mildew resistance at the *RPP8* locus of *Arabidopsis*. Plant Cell 10: 1861–1874 9811794 10.1105/tpc.10.11.1861 PMC143965

[bib24] Monot C. Penguilly D. Silué D., 2010 First confirmed report of downy mildew caused by *Hyaloperonospora parasitica* on broccoli, cauliflower and Romanesco type cauliflower heads in France. New Disease Reports 21: 5

[bib25] Monteiro A. A. Coelho P. S. Bahcevandziev K. Valério L., 2005 Inheritance of downy mildew resistance at cotyledon and adult-plant stages in ‘Couve Algarvia’ (*Brassica oleracea* var. *tronchuda*). Euphytica 141: 85–92

[bib26] Natti J. J. Atkin J. D., 1960 Inheritance of downy mildew resistance in broccoli. Phytopathology 50: 241.

[bib27] Natti J. J. Dickson M. H. Atkin J. D., 1967 Resistance of *Brassica oleracea* varieties to downy mildew. Phytopathology 57: 144–147

[bib28] O’Neill C. M. Bancroft I., 2000 Comparative physical mapping of segments of the genome of *Brassica oleracea* var. *alboglabra* that are because to sequenced regions of chromosomes 4 and 5 of *Arabidospis thaliana*. Plant J. 23: 233–243 10929117 10.1046/j.1365-313x.2000.00781.x

[bib29] Parker J. E. Coleman M. J. Szabò V. Frost L. N. Schmidt R., 1997 The Arabidopsis downy mildew resistance gene *RPP5* shares similarity to the toll and interleukin-1 receptors with N and L6. Plant Cell 9: 879–894 9212464 10.1105/tpc.9.6.879 PMC156965

[bib30] Parkin I. A. P. Sharpe A. G. Lydiate D. J., 2003 Patterns of genome duplication within the *Brassica napus* genome. Genome 46: 291–303 12723045 10.1139/g03-006

[bib31] Parkin I. A. P. Gulden S. M. Sharpe A. G. Lukens L. Trick M., 2005 Segmental structure of the *Brassica napus* genome based on comparative analysis with *Arabidopsis thaliana*. Genetics 171: 765–781 16020789 10.1534/genetics.105.042093 PMC1456786

[bib32] Ross M. T. LaBrie S. McPherson J. Stanton V. P., 1999 Screening of large-insert libraries by hybridization, pp. 5.6.1–5.6.52 Current Protocols in Human Genetics, edited by Dracopoli N. C. Haines J. L. Korf D. T. Moir C. C. Morton C. C. Wiley, New York

[bib33] Sanseverino W. Roma G. De Simone M. Faino L. Melito S., 2010 PRGdb: a bioinformatics platform for plant resistance gene analysis. Nucleic Acids Res. 38(Database issue): D814–D821 19906694 10.1093/nar/gkp978 PMC2808903

[bib34] Schranz M. E. Lysak M. A. Mitchell-Olds T., 2006 The ABC's of comparative genomics in the Brassicaceae: building blocks of crucifer genomes. Trends Plant Sci. 11: 535–542 17029932 10.1016/j.tplants.2006.09.002

[bib35] Schmidt R. Acarkan A. Boivin K., 2001 Comparative structural genomics in the Brassicaceae family. Plant Physiol. Biochem. 39: 253–262

[bib36] Yu S. Zhang F. Yu R. Zou Y. Qi J., 2009 Genetic mapping and localization of a major QTL for seedling resistance to downy mildew in Chinese cabbage (*Brassica rapa* ssp. *pekinensis*). Mol. Breed. 23: 573–590

[bib37] Sinapidou E. Williams K. Nott L. Bahkt S. Tör M., 2004 Two TIR:NB:LRR genes are required to specify resistance to *Peronospora parasitica* isolate Cala2 in Arabidopsis. Plant J. 38: 898–909 15165183 10.1111/j.1365-313X.2004.02099.x

[bib38] Soderlund C. Humphray S. Dunham A. French L., 2000 Contigs built with fingerprints, markers, and FPC V4.7. Genome Res. 10: 1772–1787 11076862 10.1101/gr.gr-1375r PMC310962

[bib39] Sulston J. Mallett F. Durvin R. Horsnell T., 1989 Image analysis of restriction enzyme fingerprint audioradiograms. Comput. Appl. Biosci. 5: 101–106 2720459 10.1093/bioinformatics/5.2.101

[bib40] Town C. D. Cheung F. Maiti R. Crabtree J. Haas B. J., 2006 Comparative genomics of *Brassica oleracea* and *Arabidopsis thaliana* reveal gene loss, fragmentation, and dispersal after polyploidy. Plant Cell 18: 1348–1359 16632643 10.1105/tpc.106.041665 PMC1475499

[bib41] Van Ooijen J. W. Voorrips R. E., 2001 *JOINMAP 3.0*, *Software for the Calculation of Genetic Linkage Maps*. Plant Research International, Wageningen, The Netherlands

[bib42] Wang J. Lydiate D. J. Parkin I. A. P. Falentin C. Delourme R., 2011 Integration of linkage maps for the Amphidiploid *Brassica napus* and comparative mapping with *Arabidopsis* and *Brassica rapa*. BMC Genomics 12: 101 21306613 10.1186/1471-2164-12-101 PMC3042011

[bib43] Yang Y. W. Lai K. N. Tai P. Y. Li W. H., 1999 Rates of nucleotide substitution in angiosperm mitochondrial DNA sequences and dates of divergence between *Brassica* and other angiosperm lineages. J. Mol. Evol. 48: 597–604 10198125 10.1007/pl00006502

[bib44] Zhang X. Wessler S. R., 2004 Genome-wide comparative analysis of the transposable elements in the related species *Arabidopsis thaliana* and *Brassica oleracea*. Proc. Natl. Acad. Sci. USA 101: 5589–5594 15064405 10.1073/pnas.0401243101 PMC397431

