# Supplementary material for: Physical Mapping in a Triplicated Genome: Mapping the Downy Mildew Resistance Locus Pp523 in Brassica oleracea L
Source: G3 (Bethesda). 2011 Dec 1;1(7):593–601. doi: 10.1534/g3.111.001099 (PMC3276173; doi:10.1534/g3.111.001099)
Supplement: Supporting Information [file supp_1_7_593__index.html]

Supporting Information 

# Physical Mapping in a Triplicated Genome: Mapping the Downy Mildew Resistance Locus *Pp523* in *Brassica oleracea* L.

## Supporting Information for Carlier *et al.*, 2011

**Files in this Data Supplement:**

- File S1 - Supporting data (.xls, 104 KB)
